# Geriatric Falls: Patient Characteristics Associated with Emergency Department Revisits

**DOI:** 10.5811/westjem.2022.6.55666

**Published:** 2022-09-12

**Authors:** Dustin D. Cox, Rachna Subramony, Ben Supat, Jesse J. Brennan, Renee Y. Hsia, Edward M. Castillo

**Affiliations:** * University of California, San Diego, School of Medicine, San Diego, California; † University of California, San Diego, Department of Emergency Medicine, San Diego, California; ‡ University of California, San Francisco, Department of Emergency Medicine, San Francisco, California; § University of California, San Francisco, Philip R. Lee Institute for Health Policy Studies, San Francisco, California

## Abstract

**Introduction:**

Falls are the leading cause of traumatic injury among elderly adults in the United States, which represents a significant source of morbidity and leads to exorbitant healthcare costs. The purpose of this study was to characterize elderly fall patients and identify risk factors associated with seven-day emergency department (ED) revisits.

**Methods:**

This was a multicenter, retrospective, longitudinal cohort study using non-public data from 321 licensed, nonfederal, general, and acute care hospitals in California obtained from the Department of Healthcare Access and Information from January 1–December 31, 2017. Included were patients 65 and older who had a fall-related ED visit identified by International Classification of Diseases codes W00x to W19x. Primary outcome was a return visit to the ED within a seven-day window following the index encounter. Demographics collected included age, gender, ethnicity/race, patient payer status, Charlson Comorbidity Index (CCI), psychiatric diagnoses, and alcohol/substance use disorder diagnoses. We performed multivariate logistic regression to identify characteristics associated with seven-day ED revisit.

**Results:**

We identified a total of 2,758,295 ED visits during the study period with 347,233 (12.6%) visits corresponding to fall-related injuries. After applying exclusion criteria, 242,572 index ED visits were identified, representing 206,612 patients. Of these, 24,114 (11.7%) patients returned to an ED within seven days (revisit). Within this revisit population, 6,161 (22.6%) presented to a facility that was distinct from their index visit, and 4,970 (18.2%) were ultimately discharged with the same primary diagnosis as their index visit. Characteristics with the largest independent associations with a seven-day ED revisit were presence of a psychiatric diagnosis (odds ratio [OR] 1.75; 95% confidence interval [CI] 1.69 to 1.80), presence of an alcohol or substance use disorder (OR 1.70; 95% CI 1.64 to 1.78), and CCI ≥ 3 (OR 2.79; 95% CI 2.68 to 2.90).

**Conclusion:**

In this study we identified 24,114 elderly fall patients who experienced a seven-day ED revisit. Patients with multiple comorbidities, a substance use disorder, or a psychiatric diagnosis exhibited increased odds of experiencing a return visit to the ED within seven days of a fall-related index visit. These findings will help target at-risk elderly fall patients who may benefit from preventative multidisciplinary intervention during index ED visits to reduce ED revisits.

## INTRODUCTION

Falls are the leading cause of traumatic injury among geriatric patients and are responsible for significant healthcare costs, loss of independence, and mortality. 1 In the United States, more than one in four adults above the age of 65 fall each year with roughly 32% resulting in serious injury. 1,2 Older adult falls have been estimated to produce an economic burden of 50 billion dollars, with Medicare bearing most of the cost. 3 The prevalence of falls and their associated costs are expected to rise with the growth of the geriatric population. One study recently demonstrated that emergency department (ED) visits for falls and fall-related injuries among the elderly increased over 27% between 2003–2010. 4 In 2019, non-fatal falls among older adults were estimated to result in nearly three million ED visits nationally. 5 As the ED increasingly plays a larger role in the care of older fall patients, there has been an effort to reduce preventable ED recidivism by identifying patients at risk of developing complications post-fall.

Prior studies suggest that a history of falling is associated with increased risk of subsequent falls, recurrent ED visits, hospitalization, and death. 6,7 Sri-on et al found more than half of elderly fall patients experienced an adverse event within six months post-fall. 6 Liu and colleagues reported that a third of geriatric patients who presented to the ED after a fall either revisited the ED or died within one year. 7 Several studies have identified an increased number of comorbidities, psychoactive drug use, and substance use order as factors associated with patients likely to revisit an ED post-fall.^6^–^9^ However, previous research exploring factors associated with fall complications has been limited by small sample sizes and an inability to examine patients across various healthcare systems, thereby limiting the generalizability of findings.

The purpose of this multicenter, retrospective cohort study was to a) characterize geriatric patients who were discharged from the ED after sustaining a fall-related ED visit, and b) identify patients at risk of returning to the ED within seven days of discharge.

## METHODS AND MATERIALS

### Study Design

This was a multicenter, retrospective, longitudinal cohort study using non-public data from 321 licensed, nonfederal, general, and acute care hospitals in California obtained from the Department of Healthcare Access and Information, formerly known as the Office of Statewide Health Planning and Development. The dataset used for this study combined the Patient Discharge Dataset and Emergency Department Dataset. This study was approved by the institution’s Human Research Protections Program.

### Study Population

The study population included patients who visited any of the 321 California nonfederal EDs from January 1–December 31, 2017. Index visits were defined as ED discharges featuring patients aged ≥65 years with a diagnosis of a fall-related injury as identified by *International Classification of Diseases, 10^th^ Revision, Clinical Modification (*ICD-10-CM) codes W00x to W19x. Multiple visits by the same patient were linked by using unique patient-record linkage numbers. We excluded patient visits without valid record linkage numbers and visits that occurred within the last seven days of the study period. Visits in which the patient was discharged to short-term general care hospitals for inpatient care, left against medical advice, died, or was sent to a psychiatric hospital were not included as index ED visits.

**Population Health Research Capsule**

What do we already know about this issue?

*Falls are the leading cause of preventable traumatic injury among elderly adults in the US, representing a significant source of morbidity and mortality.*

What was the research question?

*We wanted to identify fall patients at risk of returning to the ED within seven days of discharge.*

What was the major finding of the study?

*11.7% of elderly fall patients returned to the ED within seven days. Patients with multiple comorbidities (OR 2.79), a substance use disorder (1.70), or a psychiatric diagnosis (1.75) were more likely to return to the ED.*

How does this improve population health?

*Targeted risk assessment tools and interventions in the ED could help reduce revisits among elderly fall patients.*

### Main Outcome Measure

The primary outcome was any return visit to the ED within seven days following an index visit. Patients could have more than one seven-day ED revisit during the study period, but only one seven-day ED revisit per unique eligible index discharge was counted. However, each revisit could also be an eligible index discharge. Patient demographic variables are based upon the first ED visit within the study period and included age, gender, ethnicity/race, and expected payer. Patient-level Charlson Comorbidity Index (CCI), primary and secondary psychiatric diagnoses, and alcohol/substance use disorder diagnoses were based on all ED visits during the study period.

### Statistical Analysis

Descriptive statistics are presented as counts and percent of total by those patients with a seven-day ED revisit and those without a seven-day ED revisit. We assessed independent associations associated with a seven-day all-cause revisit after a fall-related ED visit using multivariate logistic regression. Predictors included age, gender, race/ethnicity, expected payer source, presence of a psychiatric disorder, presence of an alcohol/substance use disorder, and CCI. The most common primary diagnoses associated with seven-day ED revisits are summarized. We also report the most common conditions that make up the CCI among those with a seven-day revisit.

## RESULTS

We identified a total of 2,758,295 ED visits during the study period with 347,233 (12.6%) visits corresponding to fall-related injuries. After applying exclusion criteria, we included 242,572 index ED discharges, representing 206,612 patients. Of the 206,612 patients who were discharged from an ED following a fall-related injury, 24,114 (11.7%) returned to an ED within seven days (revisit). Among those who experienced a seven-day ED revisit, 6,161 (22.6%) returned to a facility that was distinct from their index visit and 4,970 (18.2%) were ultimately discharged with the same primary diagnosis as their index visit. Among these revisits, a total of 17,115 (62.7%) were discharged, 8,016 (29.4%) were admitted or transferred for continued care, and 1,409 (5.2%) were transferred to a skilled nursing facility, intermediate care facility, inpatient rehabilitation facility, or nursing facility.

Patient-level characteristics based on the initial visits are shown in Table 1. Patient characteristics were similar across age groups; the majority of patients were female and non-Hispanic White. Relative to those not experiencing a seven-day ED revisit, greater proportions of patients experiencing a seven-day ED revisit had a psychiatric diagnosis (50.3% vs 27.8%), alcohol or substance use disorder diagnosis (16.3% vs 8.2%), and a CCI of ≥3 (43.5% vs 22.4%).

Independent associations with having a seven-day revisit after a fall-related ED visits are reported in Table 2. Ethnicities other than Non-Hispanic White were less likely to experience a seven-day ED revisit, except non-Hispanic other/unknown, which featured no significant difference. Patients with a psychiatric diagnosis (odds ratio [OR] 1.75; 95% 1.69 to 1.80) and patients with an existing alcohol or substance use disorder (OR 1.70; 95% 1.64 to 1.78) were more likely to have a seven-day revisit after a fall-related ED visit. Patients with a CCI score of 1 (OR 1.5; 95% 1.44 to 1.570), 2 (OR 2.01; 95% 1.92 to 2.11), and ≥3 (OR 2.79; 95% 2.68 to 2.90) featured an increased odds of seven-day revisit relative to those with a score of zero.

The five most common CCI diseases associated with seven-day ED revisits are reported in Table 3. Of these, the two most common comorbidities were dementia (18.2%), followed by renal disease (16.0%). The most common diagnoses among seven-day ED revisits are reported in Table 4. Among these, the two most common diagnoses were open wound of the head (4.7%) and other septicemia (3.2%).

## DISCUSSION

Overall, we identified 206,612 geriatric patients who were discharged from an ED following a fall-related injury, 24,114 (11.7%) of whom experienced a recurrent ED visit within seven days. Of those who returned to an ED within seven days, 6,161 (22.6%) did so at a different facility than that recorded in their index visits. This indicates that single-center or single-system studies may underestimate the prevalence of geriatric falls and their sequelae. Similarly, interventions geared toward addressing recurrent geriatric falls may need to account for visits across multiple sites and multiple health systems. To reduce post-fall complications it is important to identify elderly fall patients at risk of returning to the ED prior to discharge.

The majority of elderly fall patients who had a seven-day ED revisit were women and non-Hispanic White. However, regression analysis revealed that women were less likely to experience a seven-day ED revisit. The inference here may be that while women may have increased prevalence among the geriatric fall population, individual risk for recurrent fall may in fact be higher among men than women. Our findings are consistent with prior literature demonstrating a significantly increased OR of male patients returning to the ED post-fall.^7^–^9^

Most ethnicities, compared to Non-Hispanic White, were less likely to return to the ED within seven days (Table 2). This finding is in contrast to a recent review of risk factors associated with ED recidivism, which found that ethnicity was not predictive of revisits in older adults.^9^ This review, however, included a variety of index ED visits not exclusive to falls or post-fall complications. The reason for this discrepancy is unclear; however, given the extent of our sample size it is possible that specifically fall-related revisits may be more likely in the non-Hispanic White population and present a topic for future inquiry. Age also appeared to be positively correlated with risk of return visit, which is expected given that increased age is associated with a loss of functional reserve. Our study showed a significant association between psychiatric and substance use disorder diagnoses and a seven-day ED revisit. Similar associations have been described elsewhere.^6^–^9^ Geriatric patients with psychiatric and substance use disorders are known to be at increased risk of frequent ED use, thus highlighting the importance of effective interventions to avoid potentially preventable emergencies.^10^, ^11^ There is some literature to support the use of case management interventions to reduce ED recidivism in this population.^10^ Furthermore, the increasing prevalence of geriatric EDs may improve access and referral to geropsychiatric and substance abuse resources for this group of patients.^12^

The strongest independent association for seven-day ED revisit was an increased CCI. It has been shown elsewhere that increased CCI is significantly associated with ED recidivism and death among elderly patients. 7,9 The top comorbidities reflect the chronic diseases prevalent among older US adults.^13^ According to recent data from the US Centers for Disease Control and Prevention, among adults aged ≥65 years 23.9% have one chronic disease and 63.7% have ≥2 chronic diseases.^13^ As the prevalence of older adults with comorbidities continues to grow with the aging population, proper management of existing chronic diseases may offer an approach to reduce ED recidivism among fall patients.

A recent consensus statement on geriatric fall prevention found multifactorial interventions to be most efficacious, focusing on medication review, exercise programs, and elimination of environmental hazards.^14^ This supports the importance of geriatric EDs, where several dedicated specialists such as pharmacists, physical therapists, and case managers are available to evaluate patients as needed.^12^ Also, a recent randomized control trial showed that an ED-initiated geriatric fall intervention reduced ED revisits by using pharmacists and physical therapists to assess patients prior to discharge.^15^ Lastly, close follow-up with primary care physicians has also been shown to reduced ED recidivism among the elderly.^16^

## LIMITATIONS

We obtained the data presented in this study from a statewide database in California, which has a small proportion of invalid identifiers that lack patient-level reporting from federal healthcare hospitals and does not include visit characteristics such as urgency. However, California is a diverse state representing 12% of the US population. While not wholly generalizable, the data may provide useful insight for other regions. Second, this data was limited to acute care hospitals in California; other complications necessitating other forms of less acute medical care are not represented. Lastly, it is possible that fall-related visits were not assigned the appropriate ICD 10 code, thereby resulting in underestimation of the number of fall-related index visits in this study.

## CONCLUSION

As the number of elderly fall patients continues to increase it will be vital for EDs to identify those most susceptible to a seven-day ED revisit and to meaningfully intervene prior to discharge. Within this study, patient characteristics strongly associated with a seven-day ED revisit were an existing substance use disorder diagnosis, a psychiatric diagnosis, and the presence of multiple comorbidities. More than 1/5 seven-day revisits occurred at a different facility from the index visit, highlighting the need for interventions and further study to look beyond the limits of a single center or healthcare system. Our hope is that, with the generalizability of a statewide database, these findings will help inform continued development of risk assessment tools to facilitate targeted interventions and, ultimately, reduce ED revisits among geriatric fall patients.

## Figures and Tables

**Table 1:** Patient demographics of geriatric fall patients who were initially discharged with a fall-related injury and those with a seven-day emergency department (ED) revisit and without a seven-day ED revisit.

	7-day ED revisit (n = 24,114)		No 7-day ED revisit (n = 182,498)	
Patient characteristics	Patients	%	Patients	%
Age				
65–74	7,707	32.0	65,679	36.0
75–84	8,088	33.5	61,097	33.5
≥85	8,319	34.5	55,722	30.5
Women	14,723	61.1	120,979	66.3
Race/Ethnicity				
Non-Hispanic White	16,509	68.5	119,331	65.4
Hispanic/Latino	3,823	15.9	32,054	17.6
Non-Hispanic Black	1,160	4.8	7,961	4.4
Non-Hispanic Asian/Pacific Islander	1,638	6.8	14,941	8.2
Non-Hispanic Other/Unknown	984	4.1	8,211	4.5
Psychiatric diagnosis	12,139	50.3	50,805	27.8
Substance use disorder diagnosis	3,941	16.3	15,026	8.2
Charleston Comorbidity Score				
0	5154	21.4	79,460	43.5
1	4,843	20.1	41,063	22.5
2	3,552	14.7	20,770	11.4
≥ 3	10,565	43.8	41,205	22.6

**Table 2:** Independent associations with a seven-day emergency department revisit among geriatric patients.

Patient characteristics	OR	95% CI	P-value
Age			
65–74		reference	
75–84	1.04	1.005–1.078	.025
≥85	1.07	1.03–1.11	<0.001
Women	0.88	0.86–0.91	<0.001
Race/Ethnicity			
Non-Hispanic White		Reference	
Hispanic/Latino	0.88	0.85–0.91	<0.001
Non-Hispanic Black	0.88	0.83–0.94	<0.001
Non-Hispanic Asian/Pacific Islander	0.87	0.87–0.92	<0.001
Non-Hispanic Other/Unknown	0.95	0.88–1.01	0.126
Psychiatric diagnosis	1.75	1.69–1.80	<0.001
Alcohol or substance use disorder diagnosis	1.70	1.64–1.78	<0.001
Charlson Comorbidity Score			
0		Reference	
1	1.50	1.44–1.57	<0.001
2	2.01	1.92–2.11	<0.001
≥ 3	2.79	2.68–2.90	<0.001

*OR*, odds ratio, *CI*, confidence interval.

**Table 3:** Top five Charlson Comorbidity Index diseases associated with seven-day emergency department revisits.

Disease diagnosis	N	%
Dementia	4,958	18.2%
Renal disease	4,374	16.0%
Chronic pulmonary disease	4,316	15.8%
Diabetes w/o complications	4,042	14.8%
Congestive heart failure	3,639	13.3%

**Table 4:** Top five primary diagnoses associated with seven-day emergency department revisits.

Primary diagnoses (ICD 10)	Number	%
S01 Open wound of head	1,287	4.7
A41 Other septicemia	883	3.2
S42 Fracture of shoulder and upper arm	720	2.6
S32 Fracture of lumbar spine and pelvis	716	2.6
N39 Other disorders of urinary system	697	2.6
All Other visits	22,991	84.2
Total visits	27,294	100.0

*ICD*, International Classification of Diseases 10th revision.
